# Association between Yili goose sperm motility and expression profiles of mRNA and miRNA in testis

**DOI:** 10.1186/s12864-023-09727-1

**Published:** 2023-10-24

**Authors:** Yingping Wu, Lihua Zhang, Haiying Li, Xiaoyu Zhao, Yawen Ding, Yingying Yao, Ling Wang

**Affiliations:** College of Animal Science, Xinjiang Agricultural University, Urumqi, 830000 China

**Keywords:** Yili geese, Sperm motility, Testis, miRNAs

## Abstract

**Background:**

The study was conducted to find out the candidate microRNA (miRNA) and genes that associated with sperm motility of Yili goose through small RNA sequencing of testicular tissue of Yili goose, and provide a theoretical basis for the study of the regulation mechanism of sperm motility of Yili goose gander.

**Results:**

In this study, five male geese with high sperm motility and five male geese with low sperm motility were slaughtered to obtain their testis tissues for small RNA sequencing, and biological information methods were used for data analysis. The results showed that a total of 1575 known miRNAs and 68 novel miRNAs were identified in the testis tissue of Yili goose, and 71 differentially expressed miRNAs and 660 differentially expressed genes were screened. GO functional analysis showed that miRNAs target genes were mainly involved in the binding, kinase activity, structural constituent of cytoskeleton and intermediate filament cytoskeleton. KEGG functional analysis showed that miRNAs target genes were significantly enriched in arginine and proline metabolism, glycolysis / gluconeogenesis, fructose and mannose metabolism and beta-Alanine metabolism and other pathways. miRNAs-mRNAs interaction network suggests miR-140/miR-140-3p/miR-140-3p-*NKAIN3*, let-7d-*BTG1* and miR-145-5p/miR -145a-5p-*CLEC2E* may play an important role in testis development and spermatogenesis.

**Conclusions:**

The results of this study suggest that the sperm motility of Yili goose may be regulated by different miRNAs, and the target genes are significantly enriched in pathways related to sperm metabolism, indicating that miRNAs affect the sperm motility of Yili goose by regulating the metabolic process of sperm and the expression of related genes. This study can provide a reference for revealing the regulation mechanism of Yili goose sperm motility at the molecular level.

**Supplementary Information:**

The online version contains supplementary material available at 10.1186/s12864-023-09727-1.

## Background

Yili goose is a precious poultry resource in Xinjiang, China. Compared with other domestic goose breeds in China, Yili goose has poor reproductive performance, low egg fertilization rate, and low egg production [1]. Sperm quality is an important economic characteristic of male production, and improving sperm quality is an important goal of male breeding.

The study found that the sperm motility of roosters with low reproductive performance is also low, and sperm motility can be used as a representative feature of roosters with low reproductive performance, which seriously restricts the production and development of local chicken breeds [2]. In previous studies, we found that the sperm motility of Yili geese were significant individual differences, and the sperm motility of the high sperm motility group was significantly higher than that of the low sperm motility group by 37.51% (*P* < 0.01) which seriously hindered the breeding and genetic improvement of of Yili geese [3].

Sperm motility refers to the percentage of sperm with progressive movement in semen, which is one of the most reliable indicators to evaluate the quality of semen. At the same time, the higher sperm activity also represents the higher fertilization ability of animals [4]. The normal production and motility of sperm are regulated by various factors in animals, such as hormones, growth factors and enzymes, which are the result of the joint action of complex gene regulatory networks. Over the past few years, with the development of high-throughput technology, an increasing number of studies have shown that miRNAs play a regulatory role in testicular development and spermatogenesis. Zhao et al. screened 6 differentially expressed miRNAs in the testicular tissue of white feather king pigeon. Functional enrichment analysis showed that these miRNAs were involved in serine/threonine kinase activity, ATP binding, Wnt signal pathway and MAPK signal pathway, and miR-183 was screened from the interaction network to act on *FOXO1* and play a role in sperm motility [5]. Liu et al. found 23 differentially expressed miRNAs in the testicular tissues of Beijing Fatty Chicken with high and low sperm motility, and revealed through the interaction network that gga-miR-155, gga-miR-6631-5p and gga-miR-74805p may affect the sperm viability of chicken by regulating target genes [6]. Xu et al. found that miRNA plays a crucial regulatory role in the process of spermatogenesis, in which miR-202-5p may participate in the mitotic process of spermatogenesis by targeting *LIMK2*, while miR-301a-5p regulates cell proliferation by targeting TGFβ to regulate TGFβ signaling pathway [7]. At present, there are few basic studies on goose sperm motility, especially the mechanism of miRNAs regulating sperm motility. This experiment focuses on Yili geese with extreme differences in sperm motility. Through small RNA sequencing, miRNA expression patterns were analyzed in the testis tissues of high and low sperm motility of Yili geese, and the miRNAs related to male goose sperm motility were screened and identified. This will help to better elucidate the molecular mechanism of miRNA regulation of sperm motility in Yili goose.

## Materials and methods

### Experimental animals and sample collection

In order to collect individuals with different sperm motility for sequencing, 100 ganders were trained to collect semen 2 weeks before the formal test. The semen collection method was the dorsal and abdominal massage method, and the semen collection interval was 3 days.

A total of 6 semen samples were collected in the formal experiment, with a 3-day interval between semen collection. Semen was collected using 1.5 mL preheated eppendorf tube and semen volume measured with pipette. pH value of semen Measured using a precision pH test paper with a range of 6.4 to 8.0. A small amount of semen was taken with a pipette and compared to the colorimetric strip of the test strip to determine the pH. Sperm motility, which refers to the percentage of sperm with progressive movement, was determined as follows: 10 µL of diluted semen (1:20 in 0.9% preheated Nacl) were examined under the light microscope (Olympius, Tokyo, Japan), 400 × magnification; in total 200 spermatozoa were analyzed, the percentage of sperm moving forward in a straight line was calculated. sperm concentration was calculated with hemocytometer. Count the number of sperm in five squares under a 400 × magnification light microscopy field of view. Sperm deformity rate (calculated as the percentage of abnormal spermatozoa of the 200 spermatozoa analyzed) was determined by in vivo staining with the crystal violet.

Only individuals producing ejaculates regularly should be left. The ratio of male to female geese is 1:4 for the experiment. Collect breeding eggs every day, eliminate unqualified eggs, and hatch a batch every 7 days. On the 7th day of hatching, use egg candler to illuminate the eggs and calculate the fertilization rate.

**Fig. 1:**
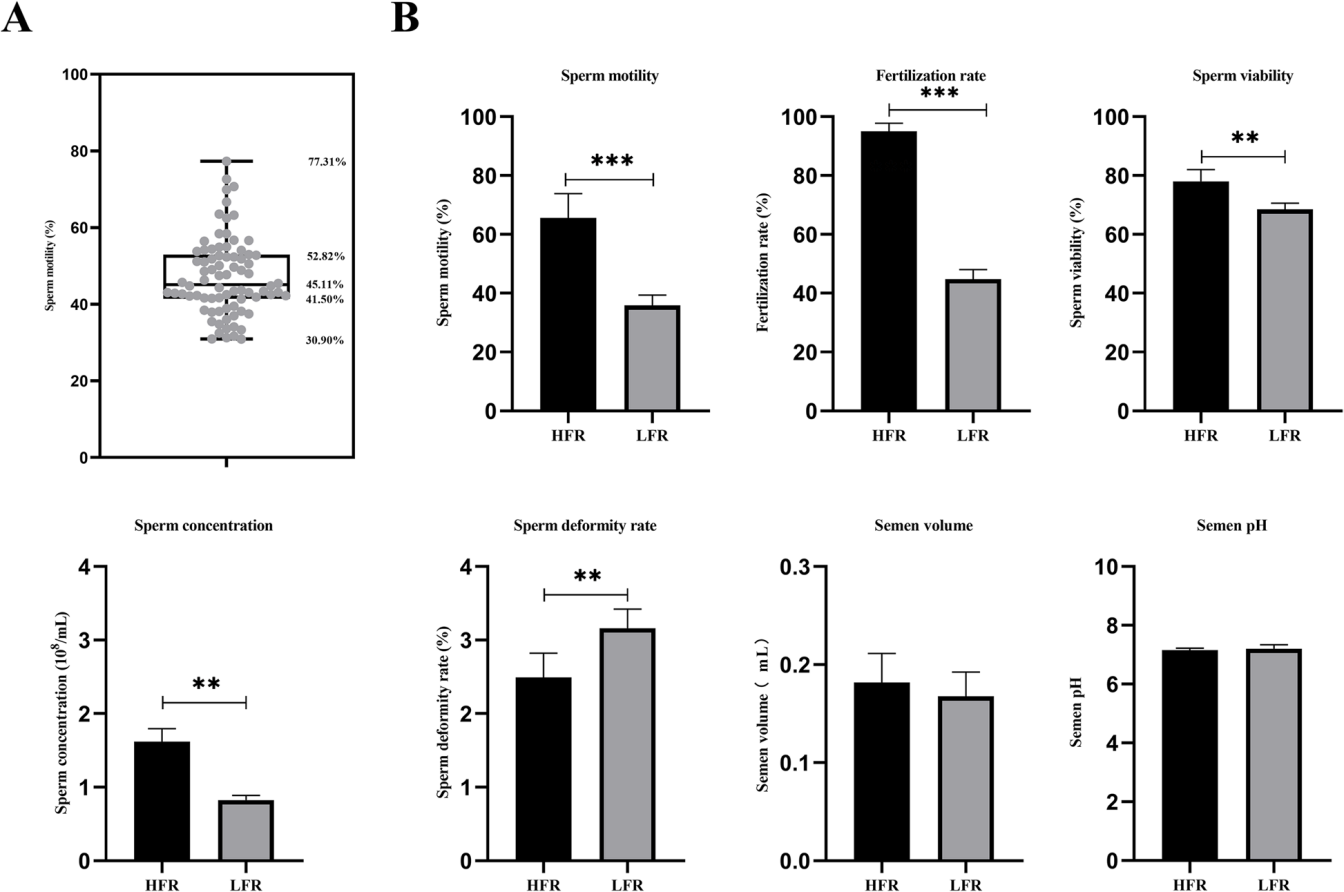
Distribution of sperm motility and semen quality. **A** Distribution of sperm motility in 100 ganders. **B** Comparison of semen quality (*N* = 10) of Yili geese between HFR group and LFR group (* indicates *P* < 0.05, ** indicates *P* < 0.01,***indicates *P* < 0.001)

Based on the semen quality and fertilization rate of 6 times, the 5 ganders with the highest sperm motility were selected as the high sperm motility group (HFR), and the 5 ganders with the lowest sperm motility were selected as the low sperm motility group (LFR). The sperm motility of the HFR group (65.63% ± 8.25%) was significantly higher than that of the LFR group (35.84% ± 3.53%) [8] (Fig. 1). The left testicular tissue was slaughtered for sequencing and stored in a refrigerator at—80 ℃. The experimental animals were provided by Hengxin Industrial Co., Ltd., Emin County, Xinjiang.

### RNA extraction, library construction and sequencing

RNA was extracted from 10 samples by Trizol method (Invitrogen, Carlsbad, CA, USA), and analyzed by agarose gel electrophoresis, Nanodrop (IMPLEN, CA, USA), and Agilent 2100 (Agilent Technologies, CA, USA). method to check its quality. Samples with RNA integrity values ≥ 8.5 were used for further analysis. After the samples passed quality control, cDNA libraries of 10 samples were constructed according to the instructions of the Illumina kit (Illuminia, San Diego, CA, USA). The llumina HiSeq2500 high-throughput sequencing platform was used for sequencing, and the sequencing read length was 150 bp for paired-end sequencing. A miRNA library of 10 samples was constructed according to the Small RNA Sample Pre Kit. Illumina SE50 sequencing was performed. The sequencing work was provided by Beijing Novogene Technology Co., Ltd.

### mRNA expression analysis

Filter raw data (raw reads), and use fastQC software [9] to evaluate the quality of sequencing data. Use the high-efficiency sequence comparison software HISAT2.0.4 [10] to compare the sequencing data of each sample with the goose reference genome [11] (https://www.ncbi.nlm.nih.gov/bioproject/589208.) for sequence alignment to obtain the position of clean reads on the reference genome and the unique sequence information of the sequenced sample. If there is no gene name, the gene ID in the reference genome is used to represent the gene name. HTSeq software [12] was used to analyze the gene expression level of each sample, and the edgeR [13] software was used to analyze the significance of expression differences. The genes with *P* < 0.05 and |log2Foldchange|> 2 were regarded as differentially expressed genes (DEGs).

### Differential expression and functional analysis of genes

In this study, GO enrichment analysis was performed on differentially expressed genes using GOseq software (Release2.12), and Pathway enrichment analysis was performed using KOBAS (v2.0) [14, 15]. GO enrichment and KEGG pathway enrichment were significantly enriched at *P* < 0.05.

### miRNA expression analysis

Filter raw data (Raw Reads), use bowtie [16] to map the screened sRNA to the reference sequence, and analyze the distribution of small RNA on the reference sequence. The miRNA prediction software miREVo [17] and mirdeep2 [18] were integrated to analyze new miRNAs, count the expression amount of known and new miRNAs in each sample, and normalize the expression amount with TPM [19]. Due to the relatively small number of miRNAs themselves and their lower expression levels compared to mRNA, differential analysis is typically performed using more lenient threshold value than mRNA. The differential expression analysis was performed with edgeR [13] based on the negative binomial distribution, and the differential expression miRNAs (DEMs) were screened according to *P* < 0.05 and |log_2_FoldChange*|*> 0. The naming of newly identified miRNAs is based on the order of the identified miRNAs.

### miRNA target gene prediction and functional annotation

Use miRanda [20] and RNAhybrid software [21] to predict target genes and target sites of differentially expressed miRNAs. In this study, the target genes of DEMs were analyzed using GOseq (Release2.12) software for GO enrichment analysis and KOBAS (v2.0) for Pathway enrichment analysis [14, 15]. GO enrichment and KEGG pathway enrichment were significantly enriched at *P* < 0.05. miRNA-mRNA interaction network analysis according to the regulatory relationship between differentially expressed miRNAs and their targets, the miRNA-mRNA interaction network was constructed and visualized using Cytoscape software [22].

### Real-time quantitative PCR verification of sequencing results

In order to verify the accuracy and reliability of the RNA-Seq results, 4 DEMs and 10 DEGs were randomly selected from the sequencing results using the RNA used for sequencing as a template. Oligo 7.0 software was used to design primers (Supplementary Table S1 and S2), and SYBR GEN reagent (TaKaRa) was used to complete the detection of the relative expression of target genes and internal reference genes in ROCHE 480 quantitative PCR instrument. Then it was detected by real-time fluorescent quantitative PCR instrument. We checked that levels of *U6* and *GAPDH* are constant in the groups HFR and LFR of testicular samples (Supplementary Fig. 1). For that purpose an additional experiment was performed in which the CT values of *U6 *(*P* = 0.744) and *GAPDH *(*P* = 0.748) did not differ between HFR group and LFR group. The reverse transcription reaction system (20 μL) included the 12.5 μL RNA-primer mix (1000ng RNA, 1μL Random6 Primer(20um), RNase free H2O Add to 12.5μL), 4 μL 5 × RT Reaction Buffer, 2μL dNTP (10 mM), 0.5 μL RNasin RNAenzyme inhibitor, 1 μL RevertAid Reverse Transcriptase.The PCR reaction system (20μL) included 10μL AceQ Universal SYBR qPCR Master Mix,0.4 uLupstream primer,0.4 uL downstream primer,2.5 μL cDNA and 6.7 μL ddH_2_O.The reaction condition was as follows: 95 °C for 5 min, followed by 40 cycles of 95 °C for 10 s, 60 °C for 30 s, Melting curve 60 ℃ to 95 ℃, with a temperature rise of 0.3 ℃ every 15 s. At least three independent biological replicates were used for each of the miRNAs and mRNA.Using *U6* and *GAPDH* as internal reference genes, the relative expression of miRNA was calculated by 2^−△△Ct^ method [23], and its log2 (fold change) value was calculated, and compared with the expression trend of transcriptome results.

## Results

### Analysis of the semen quality of Yili goose

In this study, the semen quality of 100 3-year-old Yili geese was measured in this study. According to the result of 6 times semen quality tests, the median sperm motility was 45.11%, and the top and lower quartile were 52.82% and 41.50%, respectively. Therefore, sperm motility higher than 52.82% and lower than 41.50% were considered as high and low sperm motility ganders (Fig. 1A). And 10 ganders were selected from them, 5 of which belonged to the HFR group, and the other 5 belonged to the LFR group. The sperm motility (*P* < 0.001), fertilization rate (*P* < 0.001), sperm viability (*P* = 0.003) and sperm concentration (*P* = 0.008) in the HFR group were higher than those in the LFR group, while the semen volume (*P* = 0.052) and semen pH (*P* = 0.417) do not differ (Fig. 1B).

### Summary of sequencing data of testis tissue of Yili goose

Through high-throughput sequencing, 10 samples obtained an average of 97,805,121 raw reads, and the clean reads obtained through quality control accounted for more than 99.03% of the original reads, Q20 was more than 97.49%, Q30 was more than 93.06%, and the GC content was 45.40%-47.40%, and the comparison efficiency with the reference genome is between 91.29%%-92.37%% (Supplementary Table S3).

**Fig. 2:**
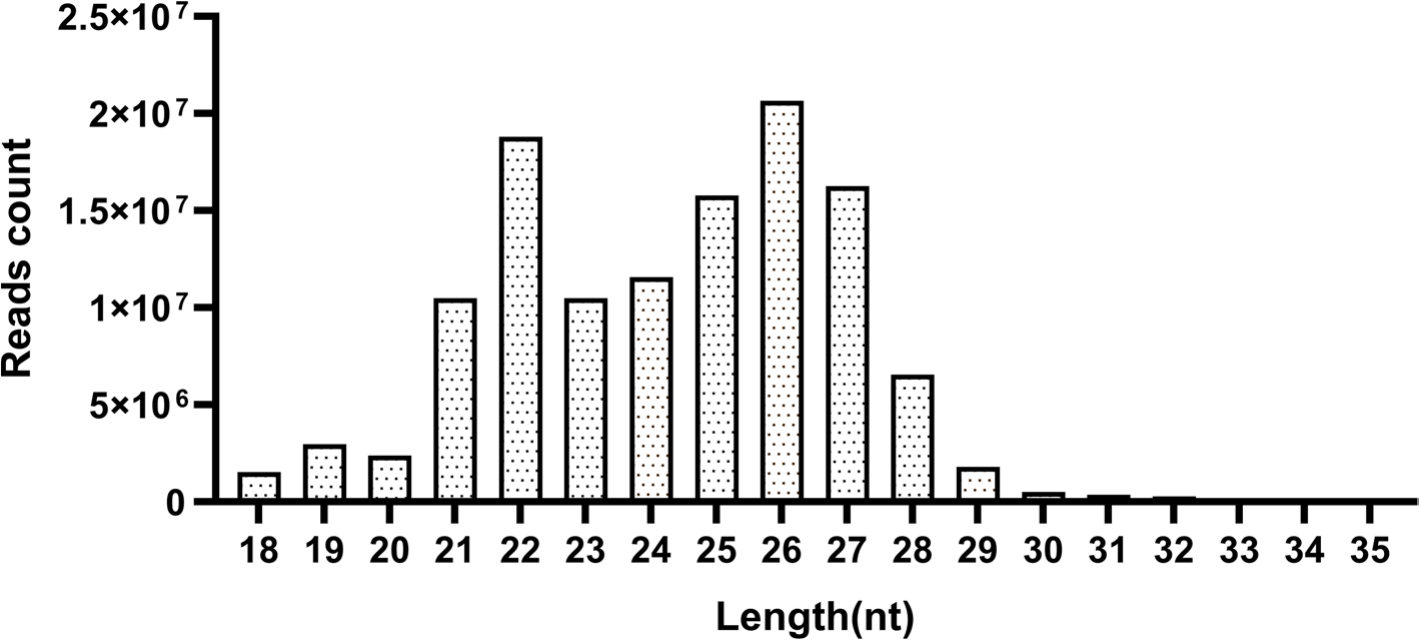
Length distribution of sRNA fragments in testis of Yili goose

Through Small RNA sequencing, 66,665,007 reads and 62,685,264 reads were obtained for Yili geese with HFR and LFR group, respectively. The clean reads obtained after quality control accounted for more than 98.53% of the original reads, and the length of the filtered sRNA fragments ranged from 21 to 27 between (Fig. 2). Each sample obtained more than 0.54G of bases, Q20 was more than 98.70%, Q30 was more than 95.51%, and the GC content was between 50.71% and 51.29%. Since there is no goose mi RBase information, bowtie [16] was used to compare the length-screened sRNA to the full miRNA library. The results show that the comparison efficiency of Reads of 10 samples and the reference genome is 89.93%. Between -92.76%% (Supplementary Table S4).

### Known miRNA and novel miRNA identification

In this study, a total of 1575 known miRNAs were identified in 10 samples (Supplementary Table S5), 877 miRNAs were co-expressed in 10 samples, 136 were specifically expressed in HFR group and 122 were specifically expressed in LFR group. A total of 68 novel miRNAs were identified in 10 samples (Supplementary Table S6). 48 miRNAs were co-expressed in 10 samples, 2 were specifically expressed in HFR group and 1 was specifically expressed in LFR group.

### Differentially expressed mRNA and miRNA

**Fig. 3:**
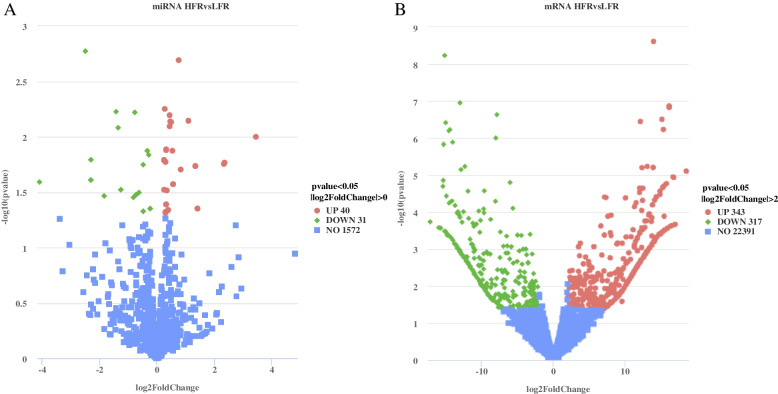
Analysis of differentially expressed mRNA and miRNA. **A** Volcano plot of differentially expressed genes; significantly upregulated genes are represented as red dots and significantly downregulated genes are represented as green dots. **B** Volcano plot of differentially expressed miRNAs

In order to explore the differences in the expression of miRNA-mRNA in the testis of ganders between the HFR group and the LFR group, *P* < 0.05 and |log_2_FoldChange|>2 were used as screening criteria during the screening of DEGs in testis tissue, and a total of 660 DEGs were screened. mRNA, of which 343 were up-regulated and 317 were down-regulated (Fig. 3A). During the screening of DEMs in testis tissue, *P* < 0.05 and |log_2_FoldChange|> 0 was used as the screening standard. A total of 71 DEMs were screened, of which 40 were up-regulated and 31 were down-regulated (Fig. 3B).

### Functional analysis of differentially expressed mRNA and miRNA target genes

**Fig. 4:**
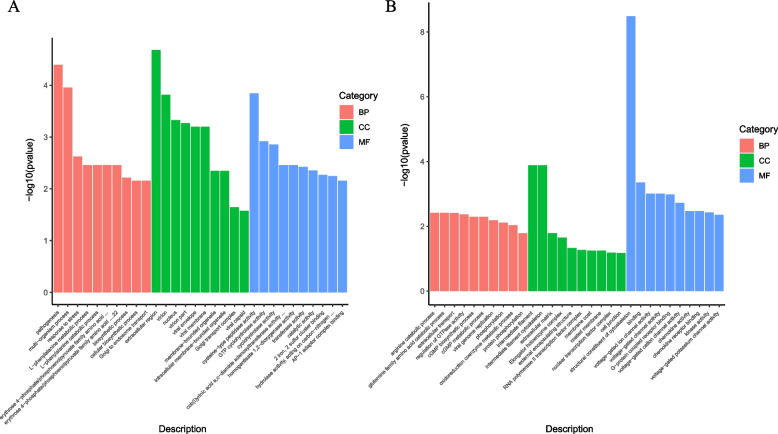
GO analysis of differentially expressed genes and target mRNAs of differentially expressed miRNAs between low and high sperm motility groups. **A** GO analysis of differentially expressed genes; BP: biological process; CC: cellular component; MF: molecular function. **B** GO analysis of target mRNAs of differentially expressed miRNAs

In order to further explore the potential regulatory mechanism of sperm motility in Yili goose, we conducted GO function enrichment analysis on the differential expression genes to further determine the important ways associated with sperm motility. The results showed that 192 of the 2386 GO terms were significantly enriched (*P* < 0.05) (Fig. 4A) (Supplementary Table S7). Differential mRNAs involved in biological processes (59.3%) were significantly enriched in pathogenesis, regulation of actin nucleationn, regulation of cell development, etc. The mRNAs involved in cell composition (14.2%) were significantly enriched in the extracellular region, nucleus and so on. The mRNAs involved in molecular function (26.3%) were significantly enriched in cysteine-type peptidase activity, GTP cyclohydrolase activity, phosphatidylinositol phosphate phosphatase activity, etc. The differential expression genes involved include spermatogenesis-associated protein *SPATA22*、*STRBP*、*SPTBN5,* testis-specific expressed protein *TEX55*、*SPOCK3,* microtubule-associated protein *MAP1A*、*MAST1,* cilia- and flagella-associated protein *CFAP69,* tight junction protein *TJB1,* etc. (Supplementary Table S7).

The target genes predicted by miRNA were enriched to 2758 GO terms, 93 of which were significantly enriched (*P* < 0.05) (Fig. 4B) (Supplementary Table S8). Target genes involved in biological processes (37.6%) were significantly enriched in arginine catabolic process, cGMP biosynthetic process, oxidoreduction coenzyme metabolic process and multicellular organismal development, etc.; target genes involved in cell composition (5.3%) were significantly enriched in intermediate filament cytoskeleton, extracellular matrix and elongator holoenzyme complex, etc.; target genes involved in molecular functions (56.9%) were significantly enriched in the structural constituent of cytoskeleton, intermediate filament, binding, and G-protein coupled receptor binding and kinase activity, etc. The target genes involved include spermatogenesis-associated protein *SPATA5,* growth factor receptor *IGF1R* and *NTRK2,* reproductive hormone receptor *PAQR9* and *GNRHR2,* myosin *MYH6、MYH7、MYO5C、MYO3B、MYO3A,* fibroblast growth factor *FGF20、FGF16、FGF4*, etc. (Supplementary Table S8).

**Fig. 5:**
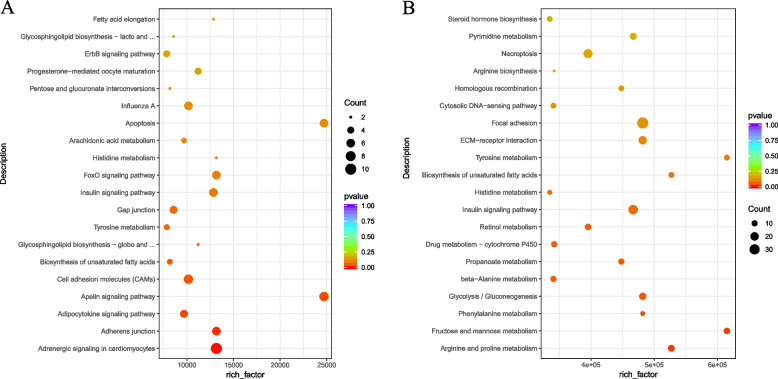
KEGG analysis of differentially expressed genes and target mRNAs of differentially expressed miRNAs between low and high sperm motility groups. **A** KEGG enrichment analysis of differential genes. The vertical axis represents the pathway name, the horizontal axis represents rich factor, the size of the point represents the number of differentially expressed genes in this pathway, and the color of the point corresponds to different *P*-value ranges. **B** KEGG enrichment analysis of target mRNAs of differentially expressed miRNAs

In order to screen important pathways associated with sperm motility, we performed KEGG pathway analysis on the target genes of differentially expressed mRNAs and miRNAs between the HFR and LFR groups. A total of 100 KEGG pathways were enriched in mRNA (Fig. 5A) (Supplementary Table S9), of which 5 pathways were significantly enriched (*P* < 0.05), including adrenergic signaling in cardiomyocytes, adherens junction, adipocytokine signaling pathway, apelin signaling pathway, and cell adhesion molecules (CAMs). Among them, Mitogen activated protein kinase 10 (*MAPK10*) participated in 21 pathways, RAC beta serine/threonine protein kinase (*AKT2*) participated in 19 pathways, Nuclear factor NF kappa B p105 subunit (*NFKB1*) participated in 13 pathways, and Adenylate cycle type 2 (*ADCY2*) participated in 11 pathways (Supplementary Table S9).

A total of 157 KEGG pathways (Fig. 5B) (Supplementary Table S10) were enriched in the target genes of miRNAs, among which 7 pathways were significantly enriched (*P* < 0.05) (Table 1). The results showed that genes associated with arginine and proline metabolism, fructose and mannose metabolism,glycolysis / gluconeogenesis, steroid hormone biosynthesis and progesterone-mediated oocyte maturation could be involved in the regulation of sperm motility (Fig. 5). The ALDH family (*ALDH3B*, *ALDH3B2*, and *ALDH9A1*) participate in at least three or more significantly enriched pathways (Table 1).

**Table 1:** KEGG pathway significantly enriched by target genes of differentially expressed miRNAs between HFR and LFR groups

KEGGE pathway	*P-Value*	Gene_names	miRNA names	KEGG ID
Arginine and proline metabolism	0.019508348	*ALDH9A1*,*DAO*,*evm.model.chr21.170*,*SRM*,*P4HA1*,*Arg2-a*,*AGMAT*,*evm.model.chr33.437*,*SMS*,*GATM*,*PYCR3*,*evm.model.chr8.559*	gga-miR-140-3p,dre-miR-145-5p,novel_133	acyg00330
Fructose and mannose metabolism	0.020220868	*TPI1*,*FBP1*,*GCK*,*ALDOB*,*HK1*,*evm.model.chr22.704*,*MPI*,*ALDOC*,*FUK*,*GMPPB*,*evm.model.ctg2812.3*	novel_133,mmu-miR-138-5p	acyg00051
Phenylalanine metabolism	0.03860189	*ALDH3B1*,*ALDH3B2*,*evm.model.chr21.170*,*AOC3*,*MIF*,*evm.model.chr33.437*,*evm.model.chr8.559*	novel_133,dre-miR-145-5p	acyg00360
Glycolysis / Gluconeogenesis	0.040381873	*ENO1*,*TPI1*,*ALDH9A1*,*ACSS2*,*FBP1*,*GCK*,*LDHB*,*ALDH3B2*,*G6PC2*,*ALDOC*,*ALDH3B1*,*ALDOB*,*ADPGK*,*HK1*	gga-miR-140-3p,novel_133,mmu-miR-138-5p	acyg00010
beta-Alanine metabolism	0.048261444	*ALDH9A1*,*ALDH3B1*,*ALDH3B2*,*ACADS*,*SRM*,*AOC3*,*ACOX1*,*SMS*,*ECHS1*,*GAD2*	gga-miR-140-3p,novel_133	acyg00410
Propanoate metabolism	0.048261444	*ACACB*,*ACSS2*,*LDHB*,*ACADS*,*MCEE*,*ACOX1*,*ACACB*,*MUT*,*ACAC*,*ECHS1*	novel_133,hsa-miR-221-3p,chi-let-7d-3p	acyg00640
Drug metabolism—cytochrome P450	0.048261444	*MGST1*,*evm.model.chr1.1295*,*FMO4*,*GSTO1*,*ALDH3B1*,*ALDH3B2*,*evm.model.chr21.170*,*evm.model.chr33.437*,*GSTM3*,*evm.model.chr8.559*	mml-miR-153–1-5p,aca-miR-129a-3p,novel_133,dre-miR-145-5p	acyg00982

### Target gene prediction and network analysis

**Fig. 6:**
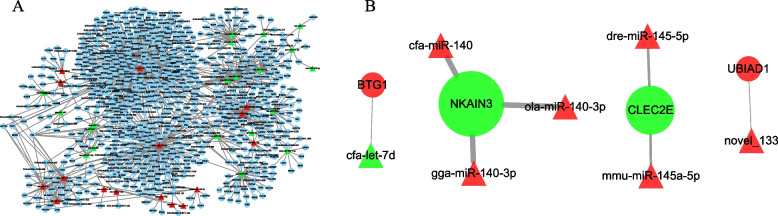
The interaction network between differentially expressed miRNAs and their target genes. **A** The interaction network between miRNAs differentially expressed in HFR and LFR groups and their target genes. **B** Four miRNA-mRNA pairs in which both partners demonstrate differential expression in HFR and LFR groups. Triangular nodes represent miRNA, and circular nodes represent target genes. Red means up, green means down. The network diagram was generated using Cytoscape

The prediction of miRNA target genes in this study is the intersection of two softwares, miRanda and RNAhybrid. This experiment uses Cytoscape software to analyze the interaction network, and the results are shown in Fig. 6. After comprehensive analysis of interaction network, a total of 71 differentially expressed miRNAs and 837 target genes were involved (Fig. 6A), of which 4 differential genes (*NKAIN3*, *BTG1*, *CLEC2E*, *UBIAD1*) could be regulated by miRNAs (Fig. 6B), a total of 7 pairs of miRNA-mRNA were differentially expressed between HFR and LFR, of which 6 pairs were negatively regulated (Fig. 6B).

### Real-time quantitative PCR verification results

**Fig. 7:**
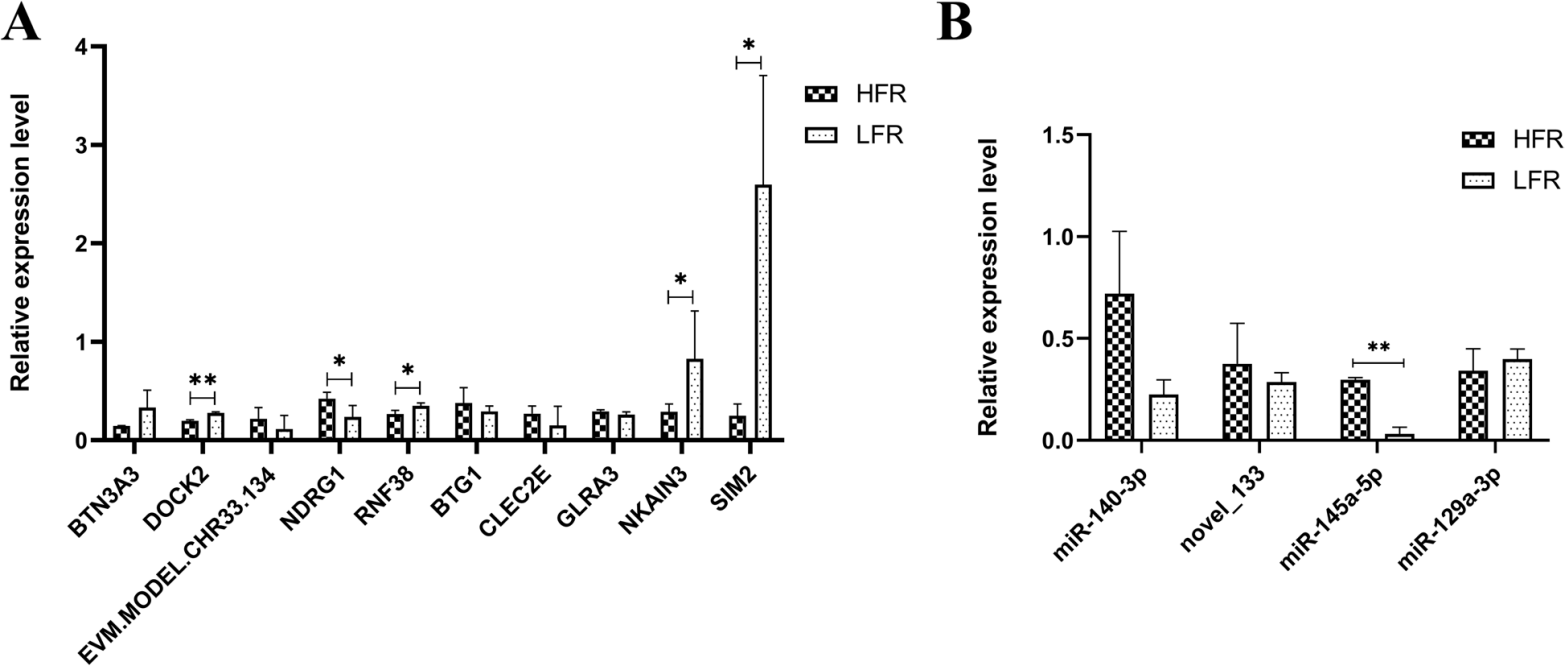
RT-qPCR a results of testicular tissue. **A** RT-qPCR analysis of differentially expressed mRNAs between HFR and LFR,The vertical axis represents the log10 value of gene expression level. **B** RT-qPCR analysis of differentially expressed miRNAs between HFR and LFR,The vertical axis represents the log10 value of miRNA expression level. (*Indicates *P* < 0.05, ** indicates *P* < 0.01)

In order to test whether the expression levels of differentially expressed mRNAs and miRNAs screened by transcriptome sequencing technology are accurate, 10 DEGs and 4 DEMs were randomly selected from transcriptome sequencing results for RT-qPCR verification. The results showed that the expression trends of the selected mRNAs and miRNAs were consistent with the transcriptome sequencing results (Fig. 7). Therefore, the results of this experiment are reliable.

## Discussion

The testicles of male birds are located in the abdominal cavity, and the main function is to produce high-quality semen [4]. The quality of male goose semen has a direct impact on reproductive performance. Sperm motility is highly positively correlated with fertility, and this trait is the most intuitive indicator in semen quality inspection [24]. Our prophase study found that the seminiferous tubules of the testes in the HFR group were larger, and the spermatogenic cells at all levels were arranged neatly and tightly, and a large number of mature sperm could be seen; while the testicular seminiferous tubules of the plow goose group with low sperm motility were relatively high. The ganders in the sperm motility group were smaller and the number of mature sperm was less [3]. It can be seen that the difference in the testis structure will also lead to the difference in the reproductive ability of birds. This experiment found that the sperm motility(*P* < 0.001), fertilization rate  (*P* < 0.001), sperm viability (*P* = 0.003) and sperm concentration (*P* = 0.008) in the HFR group were higher than that of LFR group (*P* < 0.01) (Fig. 1). This indicated that the difference in testis structure of Yili geese may further lead to the difference in sperm motility traits.In previous studies, the sperm motility of Yili geese was positively correlated with sperm viability and sperm concentration, and negatively correlated with sperm deformity rate. Ganders with high sperm motility also had better semen quality indicators [3]. Therefore, this study selected 10 individuals for sequencing based on sperm motility and fertilization rate.

The reproductive capacity of poultry has a great impact on the production efficiency of offspring, which is not only controlled by reproductive organs and reproductive cells, but also the result of the joint regulation of many genes. At present, there are a fat lot studies on the molecular mechanism of sperm motility and fertilization ability of local poultry, and most of them are concentrated on chicken, especially for ganders. More reports show that miRNA plays a vital regulatory role in normal spermatogenesis and male breeding [25–27].

In this study, ganders with great differences in sperm motility and fertilization rate were selected as the research object, and the miRNAs and mRNA gene pairs in the testes of Yili goose were identified by comprehensive analysis of miRNA and mRNA transcripts. The results of the study help to better understand the underlying mechanisms regulating sperm motility traits in Yili geese and reveal candidate molecular markers for the reproductive performance of Yili geese. In this study, the testis tissues of high and low sperm motility Yili geese were sequenced by small RNA sequencing, and more than 98.53% of the clean reads were obtained, and 89.93%-92.76% of the clean reads could be compared to the goose reference genome (Supplementary Table S1), indicating that the sequencing data in this study were of high quality. The length distribution of sRNA fragments in the testis tissue of Yili goose showed that the length of the filtered sRNA fragments was between 21 and 27 nt, and the proportion of 22 nt and 26 nt was the highest (Fig. 2), slightly higher than the normal size of miRNAs [28].

In this study, 660 differential mRNAs (*P* < 0.05, |log_2_Foldchange|≥ 2), 1575 known miRNAs and 68 novel miRNAs were identified, and 71 differentially expressed miRNAs were screened (*P* < 0.05) (Fig. 3). Among them, miR-7–1-3p, miR-184, and miR-184b-5p had the largest up-regulation multiples of miRNAs, and the differential multiples were 3.46, 2.36, and 2.35 times, respectively. The miRNAs included miR-153–1-5p, miR-375-3p and miR-206-3p, and the differences were 4.09-fold, 2.48-fold and 2.30-fold, respectively. These miRNAs with large fold difference may have potential regulatory roles in sperm motility traits. For example, miR-7–1-3p can be used as a diagnostic biomarker for non-obstructive azoospermia [29]. miR-184 is located in the germ cells of mouse testis, and its expression level increases with age, and miR-184 may be involved in the post-transcriptional regulation of Ncor2 and other genes during mouse spermatogenesis [30]. Guo et al. found that miR-375 inhibited the proliferation of pig testicular Sertoli cells, and miR-375 down-regulated its target genes RLF and *HIGD1A* at the mRNA and protein expression levels, indicating that miR-375 can target RLF and *HIGD1A* affect the development of testicular tissue [31]. Therefore, the differentially expressed miRNAs found in this study may play a key role in the sperm motility of Yili geese.

In order to explore the relationship between these differentially expressed miRNAs and the sperm motility of Yili goose, GO enrichment analysis was performed on the target genes of these miRNAs (Supplementary Table S8). It was found that they were mainly involved in molecular functions (56.9%), and were significantly enriched in the structural constituent of cytoskeleton, intermediate filament and binding process. The eukaryotic cytoskeleton is defined as composed of actin filaments, microtubules, intermediate filaments, and septicin proteins [32], and is an integral part of the process of spermatogenesis, in motility, intracellular transport, differentiation and play a key role in cell division. The cytoskeleton can directly promote the mitosis and meiosis of spermatogenesis. The unique cytoskeleton structure is an integral part of sperm cell remodeling and sperm function, and is crucial to the reproductive performance of male animals [33].

The top 5 pathways significantly enriched by KEGG are Arginine and proline metabolism, Fructose and mannose metabolism, Phenylalanine metabolism, and glycolysis /glycolysis / gluconeogenesis and beta-Alanine metabolism (Supplementary Table S10). These pathways are involved in sperm metabolism. Arginine is one of the basic amino acids that make up sperm nucleoprotein [34], and is also the only substrate for the production of NO in the body. NO regulates sperm motility by participating in the regulation of testicular circulation. Arginine has been proved to be able to maintain and improve sperm motility [35]. Sperm motility, capacitation and acrosome reaction are cell events with high energy consumption. In order to perform these (and other) physiological functions, sperm needs more adenosine triphosphate (ATP) than other cells [36, 37]. It is very important to maintain the energy state of cells for sperm motility [38]. Studies have shown that most mammals can produce ATP through glycolysis, the tricarboxylic acid cycle, and the oxidation of fatty acids and ketone bodies [39]. The movement of sperm mainly depends on the swing of the main flagellum segment, which is rich in glycolysis enzymes [40]. β-alanine generates energy by participating in the tricarboxylic acid cycle [38]. Sperm can also metabolize fructose through the glycolytic pathway. Fructose is abundant in poultry seminal plasma and plays a crucial role in maintaining sperm motility and seminal plasma osmotic pressure [41, 42]. The above studies suggest that amino acids participate in the cell metabolism of motile sperm, are energy supplement sources, and can enhance sperm motility. The results of this study showed that significantly enriched genes related to amino acid metabolism, glycolysis, gluconeogenesis, and fructose and mannose metabolism, miRNA and KEGG pathways played crucial roles in the regulation of sperm metabolism and energy production. In summary, these findings suggest that molecules controlling sperm metabolism may affect sperm motility in ganders.

Predicting the mRNA targets of miRNAs will help characterize the miRNAs involved in biological processes and the molecular mechanisms by which mRNAs function. In this study, it was found that 4 differential genes (*NKAIN3*, *BTG1*, *CLEC2E*, *UBIAD1*) could be regulated by miRNA (Fig. 6B), and a total of 6 pairs of miRNA-mRNA were differentially expressed between HFR and LFR, and were negatively regulated (Fig. 6B). Among them, *NKAIN3* (Sodium/Potassium Transporting ATPase Interacting 3) was negatively correlated with 3 differential miRNAs. *NKAIN3* is one of the members of the NKAIN (Na,K-Atpase INteract-ing) protein family and was found to interact with the β1 subunit of Na,K-ATPase (NKA) [43]. Na, K ATPase (NKA) is a kind of transmembrane carrier protein widely present in the cell membrane of eukaryotic organisms. It is an important system to maintain the transport of Na^+^and K^+^ions and energy metabolism in cells, and directly or indirectly participates in the ion regulation inside and outside cells, physiological activities of the body and thermoregulation process [44]. Similar to somatic cells, sperm also maintain a transmembrane gradient of Na ^+^ and K^+^ [45]. It has been shown that multiple NKA subunits were found in chicken and mouse sperm membranes through proteomic studies [46, 47], prove that NKA plays a role in sperm fertilization. B-cell translocation gene 1 (*BTG1*), a member of the TOB/BTG protein family, is an antiproliferative gene. *BTG1 *protein products are involved in various cellular processes such as cell division, DNA repair, transcriptional regulation, and messenger RNA stability [48]. Raburn [49] etc. determined the level of *BTG1* in rat testis tissue. The results of Northern blot showed that *BTG1* was expressed in both meiotic and early meiotic germ cells of adult rat testes. In situ hybridization showed that the expression of *BTG1* was stage-dependent, with *BTG1* transcripts being the highest in round sperm cells, followed by pachytene spermatocytes, and almost undetectable in spermatids, suggesting that *BTG1* transcripts in spermatids play a role in the occurrence. In this study, *BTG1* was up-regulated in LFR, suggesting that it may be involved in regulating the cell division of germ cells and function as an anti-proliferation factor.The results of our experiments indicated that *BTG1* was up-regulated in the ovaries of HEP geese.

*CLEC2E* (C-type lectin domain family 2 member E) is a homodimeric cell surface glycoprotein, and in mice, the expression of *CLEC2E* is highly restricted to the gastrointestinal tract. The expression of ClR-a (*CLEC2E*) in intestinal epithelial cells is downregulated under inflammatory conditions, which may contribute to the suppression of mucosal immune responses [50]. Woo et al. [51] compared the genes expressed in intestinal epithelial cells from germ-free (GF) and conventionally reared (CNV) mice by RNA sequencing, and determined that the C-type lectin 2 member e (*CLEC2E*; Clr-a) was one of the most significantly downregulated genes in intestinal epithelial cells (IECS) in response to microbial exposure. Using gut organoid cultures, we determined that the microbiota directly affects the intrinsic defenses of IECs and found that down-regulation of the microbiota on *CLEC2E* reduced the colonization of pathogens. In this study, *CLEC2E* was down-regulated in testicular tissue and involved in GO terminology related to virus reproduction and transmission (Supplementary Table S8), indicating that *CLEC2E* is not a gastrointestinal specific gene in Yili goose, and it can prevent pathogen invasion during sperm formation. thus playing an important protective role. In this study, *NKAIN3*, *BTG1* and *CLEC2E* were predicted as targets of miR-140-3p, let-7d and miR-145a-5p, respectively.

## Conclusion

In summary, this study established the expression patterns of genes and miRNAs in the testis of Yili geese in the high sperm motility group and low sperm motility group by transcriptome sequencing technology. A total of 660 genes and 71 miRNAs were differentially expressed between the two groups. GO and KEGG analysis showed that these miRNAs were involved in the structural constituent of cytoskeleton, intermediate filament, arginine and proline metabolism, fructose and mannose metabolism, glycolysis / gluconeogenesis and other signaling pathways.

The miRNA-mRNA interaction network suggests miR-140-3p-*NKAIN3*, let-7d-*BTG1* and miR-145-5p-*CLEC2E* may play an important role in testis development and spermatogenesis. This study provides more clues for further research on the role of miRNAs-genes in the sperm motility of Yili goose. However, the involvement of miRNA in testicular development and sperm motility in Yili geese through targeted genes remains to be further validated.

### Supplementary Information

**Additinal file 1: Supplementary Table S1.** RT-PCR primers of the differentially expressed miRNAs.

**Additional file 2: Supplementary Table S2.** RT-PCR primers of the differentially expressed genes.

**Additional file 3: Supplementary Figure 1.** CT values of different internal reference genes in the testicular tissue of Yili geese . The horizontal axis represents different reference genes, while the vertical axis represents the CT value of the reference genes.ns indicates *P*>0.05.

**Additional file 4: Supplementary Table S3.** mRNA sequencing data quality.

**Additional file 5: Supplementary Table S4.** miRNA sequencing data quality.

**Additional file 6: Supplementary Table S5.** Know miRNA.

**Additional file 7: Supplementary Table S6.** Novel miRNA.

**Additional file 8: Supplementary Table S7.** GO analys of differentially expressed genes between HFR and LFR groups.

**Additional file 9: Supplementary Table S8.** GO analysis of target mRNAs of differentially expressed miRNAs between HFR and LFR groups.

**Additional file 10: Supplementary Table S9.** KEGG analysis of differentially expressed genes between HFR and LFR groups.

**Additional file 11: Supplementary Table S10.** KEGG analysis of target mRNAs of differentially expressed miRNAs between HFR and LFR groups.

## Data Availability

The data presented in the study are deposited in the BioProjectin NCBI repository (https://www.ncbi.nlm.nih.gov/bioproject/PRJNA856143) and (https://www.ncbi.nlm.nih.gov/bioproject/PRJNA914617), accession number is PRJNA856143 and PRJNA914617.
