# Supplementary material for: Association between Yili goose sperm motility and expression profiles of mRNA and miRNA in testis
Source: BMC Genomics. 2023 Oct 24;24:640. doi: 10.1186/s12864-023-09727-1 (PMC10599010; doi:10.1186/s12864-023-09727-1)
Supplement: Supplementary file 1 — Additinal file 1: Supplementary Table S1. RT-PCR primers of the differentially expressed miRNAs. [file 12864_2023_9727_MOESM1_ESM.docx]

##### Supplementary Table S1 RT-PCR primers of the differentially expressed miRNAs

| miRNA | Forward primer | Reverse primer |
| --- | --- | --- |
| gga-miR-140-3p | CGCGCCACAGGGTAGAAC | CAGTGCAGGGTCCGAGGTAT |
| novel_133 | CGTGCAGGATGTCTGGGTG | CAGTGCAGGGTCCGAGGTAT |
| mmu-miR-145a-5p | GGCGGTCCAGTTTTCCCAG | CAGTGCAGGGTCCGAGGTAT |
| aca-miR-129a-3p | CGGGAAGCCCTTACCCCAA | CAGTGCAGGGTCCGAGGTAT |
| U6 | GGAACGATACAGAGAAGATTAGC | CGGAACGCTTCACGAATTTG |
