# Supplementary material for: Association between Yili goose sperm motility and expression profiles of mRNA and miRNA in testis
Source: BMC Genomics. 2023 Oct 24;24:640. doi: 10.1186/s12864-023-09727-1 (PMC10599010; doi:10.1186/s12864-023-09727-1)
Supplement: Supplementary file 2 — Additional file 2: Supplementary Table S2. RT-PCR primers of the differentially expressed genes. [file 12864_2023_9727_MOESM2_ESM.docx]

Supplementary Table S2 RT-PCR primers of the differentially expressed genes

| Gene | Primer Sequence | Product Length | Annealing Temperature |
| --- | --- | --- | --- |
| BTN3A3 | F:GGACCAGGAGGGGTTCTTTG | 139 bp | 60.0℃ |
|  | R:GAAGAAGGGAGCTGCGATGT |  |  |
| Ndrg1 | F:AGTGCCCGTGCTTTCTGTAA | 106 bp | 60.2℃ |
|  | R:TCTCTAGGTCTCGCCGACTG |  |  |
| DOCK2 | F:GCCCTGTGTCACCTGATGAA | 166 bp | 60.0℃ |
|  | R:TCCACAGAGTGCTTTCCGTC |  |  |
| RNF38 | F:AGCCTTTCACCCTCCGAATG | 413 bp | 60.0℃ |
|  | R:GAGCCACCGTTGTCACTGTA |  |  |
| chr33.134 | F:CTACGGAGTGGTGCTCTTTGG | 124 bp | 60.7℃ |
|  | R:TCCGTTGTTGGCTATGGCAG |  |  |
| NKAIN3 | F:GCTGCATACGAAGGGTGGT | 76 bp | 60.1℃ |
|  | R:CAGCCTGTCACAGAGACGTAG |  |  |
| BTG1 | F:AGGTGCCCTCTTCGGATAAGT | 78 bp | 60.3℃ |
|  | R:TTTCCGCCAAGCTATGCTCA |  |  |
| SIM2 | F:AGAACAACGCCTTACCCACA | 172 bp | 59.9℃ |
|  | R:GCAAGCTATATGACGGGGCA |  |  |
| Glra3 | F:ACCGAGTGAACATCTTTCTCCG | 131 bp | 60.0℃ |
|  | R:AGCAAAGAACAAATCAGGTTTCCA |  |  |
| Clec2e | F:CCTTCAACACCTGGTTTGAGATG | 127 bp | 59.7℃ |
|  | R:AGGACGTAGTTGTTGGGTCTG |  |  |
| GAPDH | F:TGATGCTCCCATGTTCGTGATG | 168 bp | 60.7℃ |
|  | R:GTGATGGCATGGACAGTGGT |  |  |

Note: F: for upstream primer R: for downstream primer
