# Supplementary material for: Association between Yili goose sperm motility and expression profiles of mRNA and miRNA in testis
Source: BMC Genomics. 2023 Oct 24;24:640. doi: 10.1186/s12864-023-09727-1 (PMC10599010; doi:10.1186/s12864-023-09727-1)
Supplement: Supplementary file 3 — Additional file 3: Supplementary Figure 1. CT values of different internal reference genes in the testicular tissue of Yili geese . The horizontal axis represents different reference genes, while the vertical axis represents the CT value of the reference genes.ns indicates P>0.05. [file 12864_2023_9727_MOESM3_ESM.doc]

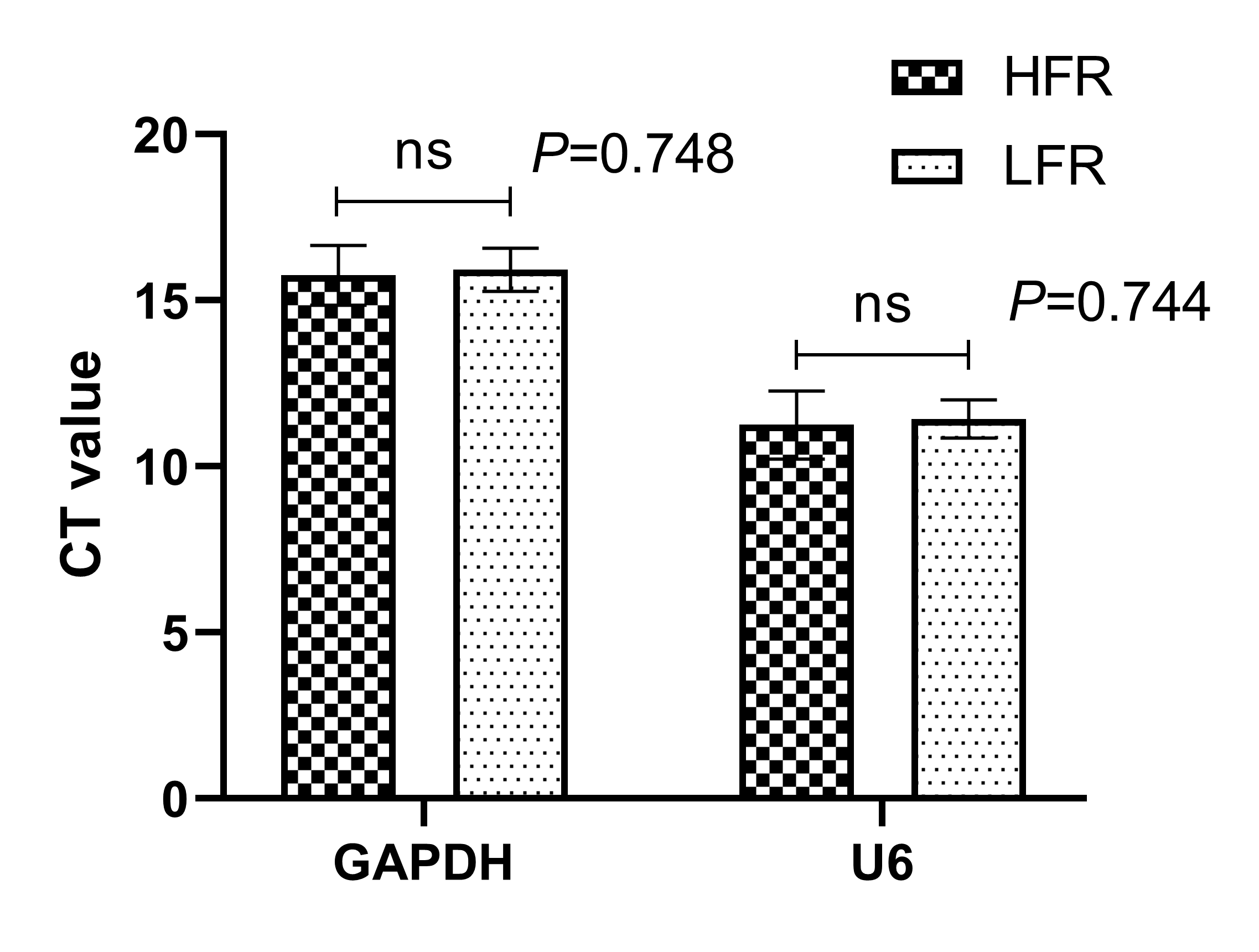


Supplementary Figure 1 CT values of different internal reference genes in the testicular tissue of Yili geese . The horizontal axis represents different reference genes, while the vertical axis represents the CT value of the reference genes.ns indicates *P*>0.05
