# Supplementary material for: Association between Yili goose sperm motility and expression profiles of mRNA and miRNA in testis
Source: BMC Genomics. 2023 Oct 24;24:640. doi: 10.1186/s12864-023-09727-1 (PMC10599010; doi:10.1186/s12864-023-09727-1)
Supplement: Supplementary file 4 — Additional file 4: Supplementary Table S3. mRNA sequencing data quality. [file 12864_2023_9727_MOESM4_ESM.doc]

##### Supplementary Table S3 mRNA sequencing data quality

| **Sample** | **Raw Reads** | **Clean reads** | **Bases** | **Error rate** | **Q20** | **Q30** | **GC content** | **Total mapped** |
| --- | --- | --- | --- | --- | --- | --- | --- | --- |
| HFR_1 | 86345158 | 85695512  (99.25%) | 12.85 | 0.03 | 97.65 | 93.43 | 47.19 | 78316871 (91.39%) |
| HFR_2 | 105769898 | 104987610  (99.26%) | 15.75 | 0.03 | 97.63 | 93.38 | 46.50 | 96332847 (91.76%) |
| HFR_3 | 93937816 | 93265686  (99.28%) | 13.99 | 0.03 | 97.49 | 93.06 | 46.53 | 85686995 (91.87%) |
| HFR_4 | 105232800 | 104604358  (99.40%) | 15.69 | 0.03 | 97.63 | 93.39 | 46.81 | 96203761 (91.97%) |
| HFR_5 | 107655208 | 106848128  (99.25%) | 16.03 | 0.03 | 97.61 | 93.36 | 47.22 | 98144265 (91.85%) |
| LFR_1 | 104869524 | 103850998  (99.03%) | 15.58 | 0.03 | 97.6 | 93.37 | 47.40 | 95013805 (91.49%) |
| LFR_2 | 82214726 | 81594444  (99.25%) | 12.24 | 0.03 | 97.68 | 93.48 | 46.60 | 74489752 (91.29%) |
| LFR_3 | 106378240 | 105732972  (99.39%) | 15.86 | 0.03 | 97.6 | 93.28 | 45.40 | 97160534 (91.89%) |
| LFR_4 | 84278058 | 83586102  (99.18%) | 12.54 | 0.03 | 97.53 | 93.11 | 45.80 | 77212079 (92.37%) |
| LFR_5 | 101369790 | 100638104  (99.28%) | 15.1 | 0.03 | 97.6 | 93.28 | 46.65 | 92182939 (91.6%) |
