# Supplementary material for: Association between Yili goose sperm motility and expression profiles of mRNA and miRNA in testis
Source: BMC Genomics. 2023 Oct 24;24:640. doi: 10.1186/s12864-023-09727-1 (PMC10599010; doi:10.1186/s12864-023-09727-1)
Supplement: Supplementary file 5 — Additional file 5: Supplementary Table S4. miRNA sequencing data quality. [file 12864_2023_9727_MOESM5_ESM.doc]

##### Supplementary Table S4 miRNA sequencing data quality

| **Sample** | **Raw Reads** | **Clean reads** | **Bases** | **Error rate** | **Q20** | **Q30** | **GC content** | **Mapped sRNA** |
| --- | --- | --- | --- | --- | --- | --- | --- | --- |
| HFR_1 | 12808015 | 12664841 (98.88%) | 0.640G | 0.01% | 99.16% | 96.61% | 50.88% | 11054868 (92.38%) |
| HFR_2 | 15363252 | 15227986 (99.12%) | 0.768G | 0.01% | 99.49% | 97.91% | 50.80% | 13283168 (91.29%) |
| HFR_3 | 13103895 | 12977164 (99.03%) | 0.655G | 0.01% | 98.92% | 96.02% | 50.81% | 11427508 (91.95%) |
| HFR_4 | 14667180 | 14518235 (98.98%) | 0.733G | 0.01% | 99.09% | 96.39% | 50.93% | 12840779 (92.27%) |
| HFR_5 | 10722665 | 10616225 (99.01%) | 0.536G | 0.01% | 98.70% | 95.51% | 50.83% | 9360215 (92.04%) |
| LFR_1 | 12094047 | 11916199 (98.53%) | 0.605G | 0.01% | 98.81% | 96.17% | 50.71% | 10396279 (92.39%) |
| LFR_2 | 11762723 | 11629579 (98.87%) | 0.588G | 0.01% | 98.97% | 96.06% | 50.81% | 9996704 (89.93%) |
| LFR_3 | 14077229 | 13880227 (98.60%) | 0.704G | 0.01% | 98.98% | 96.17% | 51.16% | 12151592 (91.83%) |
| LFR_4 | 13487920 | 13319324 (98.75%) | 0.674G | 0.01% | 98.95% | 95.95% | 51.29% | 11187512 (92.70%) |
| LFR_5 | 11263345 | 11148060 (98.98%) | 0.563G | 0.01% | 98.96% | 96.48% | 51.05% | 9295789 (92.76%) |
